# Nuclear translocation of vitellogenin in the honey bee (*Apis mellifera*)

**DOI:** 10.1007/s13592-022-00914-9

**Published:** 2022-03-15

**Authors:** Heli Salmela, Gyan P. Harwood, Daniel Münch, Christine G. Elsik, Elías Herrero-Galán, Maria K. Vartiainen, Gro V. Amdam

**Affiliations:** 1 Organismal and Evolutionary Biology Research Program, University of Helsinki, Viikinkaari 1, 00014 Helsinki, FI Finland; 2 Department of Entomology, University of Illinois at Urbana-Champaign, 320 Morrill Hall 505 South Goodwin Avenue, Urbana, IL 61801 USA; 3 Faculty of Environmental Sciences and Natural Resource Management, Norwegian University of Life Sciences, N-1432 Aas, Norway; 4 Division of Animal Sciences, University of Missouri, S108 Animal Sciences Research Center (ASRC), Colombia, MO 65211 USA; 5 Centro Nacional de Biotecnología, Calle Darwin 3, 28049 Madrid, Spain; 6 Institute of Biotechnology, Helsinki Institute of Life Science, University of Helsinki, Viikinkaari 5, 00014 Helsinki, Finland; 7 School of Life Sciences, Arizona State University, 427 East Tyler Mall, Tempe, AZ 85281 USA

**Keywords:** Vitellogenin, nuclear protein, DNA binding

## Abstract

Vitellogenin (Vg) is a conserved protein used by nearly all oviparous animals to produce eggs. It is also pleiotropic and performs functions in oxidative stress resistance, immunity, and, in honey bees, behavioral development of the worker caste. It has remained enigmatic how Vg affects multiple traits. Here, we asked whether Vg enters the nucleus and acts via DNA-binding. We used cell fractionation, immunohistology, and cell culture to show that a structural subunit of honey bee Vg translocates into cell nuclei. We then demonstrated Vg-DNA binding theoretically and empirically with prediction software and chromatin immunoprecipitation with sequencing (ChIP-seq), finding binding sites at genes influencing immunity and behavior. Finally, we investigated the immunological and enzymatic conditions affecting Vg cleavage and nuclear translocation and constructed a 3D structural model. Our data are the first to show Vg in the nucleus and suggest a new fundamental regulatory role for this ubiquitous protein.

**Supplementary information:**

The online version contains supplementary material available at 10.1007/s13592-022-00914-9.

## Introduction

Vitellogenin (Vg) is an egg yolk precursor protein common to nearly all oviparous animals and likely evolved around 700 million years ago as the oldest member of the large lipid transfer protein family (Hayward et al. 2010). It is highly pleiotropic, functioning as a storage protein (Amdam and Omholt 2002), an immunomodulator (Du et al. 2017; Garcia et al. 2010; Li et al. 2008), an antioxidant (Havukainen et al. 2013; Salmela et al. 2016), and a behavior regulator (Amdam et al. 2006; Antonio et al. 2008; Nelson et al. 2007). These functions are observed in taxa as disparate as fish, corals, and insects, suggesting that Vg may have evolved to support multiple functions. Honey bees (*Apis mellifera*) have been the premier insect model for studying Vg pleiotropy, owing largely to the central role it plays in colony division of labor. Here, the non-reproductive worker caste performs a series of age-dependent tasks that is regulated, in part, by Vg titers circulating in their hemolymph (Nelson et al. 2007).

The cause of Vg’s pleiotropy remains somewhat enigmatic. We have detailed understanding of how Vg forms egg yolk (Tufail and Takeda 2008), and how it acts in innate immunity as a pathogen pattern recognition receptor (Li et al. 2008), but the molecular mechanism(s) by which it influences multiple traits remains unclear. Some pleiotropic proteins implement their multiple effects by acting as transcription factors via DNA-binding or participating in gene-regulatory complexes that affect the expression of many genes (Chesmore et al. 2016). Interestingly, downregulation of honey bee Vg by means of RNA-interference–mediated *vg* gene knockdown alters the expression of thousands of genes (Wheeler et al. 2013). However, no previous research has addressed or established whether Vg can translocate into the cell nucleus and bind DNA. This lack of investigation may be due to Vg’s large size, as the proteins are typically around 200 kDa (Tufail and Takeda 2008), while most passively transported nuclear proteins are below 60 kDa (Wang and Brattain 2007). Yet in many animals, Vg is enzymatically cleaved and reassembled prior to secretion (Tufail and Takeda 2008). In invertebrates such as insects, Vg is cleaved in the vicinity of a multiple-phosphorylated polyserine linker sequence stretch that resides between two evolutionarily conserved Vg protein domains called the N-sheet and the α-helical domain (Baker 1988; Havukainen et al. 2012; Tufail and Takeda 2008). In honey bees, this cleavage occurs in vivo in the fat body, the primary production site of Vg (Tufail and Takeda 2008), and results in a detached 40-kDa N-sheet (Havukainen et al. 2011, hereafter termed "Vg N-sheet" for clarity). The function of this Vg N-sheet is not fully known, but in fish, it serves as the receptor binding region (Li et al. 2003).

In this study, we asked whether pleiotropic effects of Vg may be partly explained by nuclear translocation and DNA-binding of the conserved Vg N-sheet, and we used the honey bee as our study organism. First, we used Western blots of the fat body nucleoplasm and confocal microscopy of fat body cells to test whether the Vg N-sheet can translocate into cell nuclei, using an antibody targeting the Vg N-sheet. We verified these results by observing nuclear uptake of fluorescently labelled honey bee Vg in an insect cell culture. Second, we predicted the DNA-binding capability of the Vg N-sheet using sequence- and structure-based software, and then used ChIP-seq to identify Vg N-sheet binding sites in honey bee DNA. As honey workers’ tasks and Vg titers covary with age, we compared workers that were 1 day old (< 24 h since emergence) with those 7 days old to determine whether Vg N-sheet-DNA binding sites persist as workers transition between behavioral tasks. We searched for de novo binding motifs and performed a gene ontology analysis of Vg N-sheet-DNA binding sites, identifying many sites associated with immune- and behavior-related genes. These data motivated a functional response-to-challenge assay using *Escherichia coli* to reveal if behavior of Vg cleavage and translocation is, in fact, dynamic. Finally, we established the enzymatic conditions required for Vg cleavage, and we developed a three-dimensional structure model to elucidate the critical N-terminal area of the protein.

## Material and methods

### Antibody against honey bee Vg N-sheet

The 40-kDa honey bee Vg N-sheet (Uniprot ID Q868N5; the amino acid residues 24–360) was produced in *E. coli* by GenScript (Piscataway, NJ, USA); it was subcloned into pUC57 vector, and an N-terminal hexahistidine tag was used for one-step affinity purification. The polyclonal serum antibody was raised in a rat by Harlan Laboratories (Boxmeer, the Netherlands) and tested by Western blotting against proteins extracted from the honey bee (see S1).

### Cell fractionation

**Figure 1.**
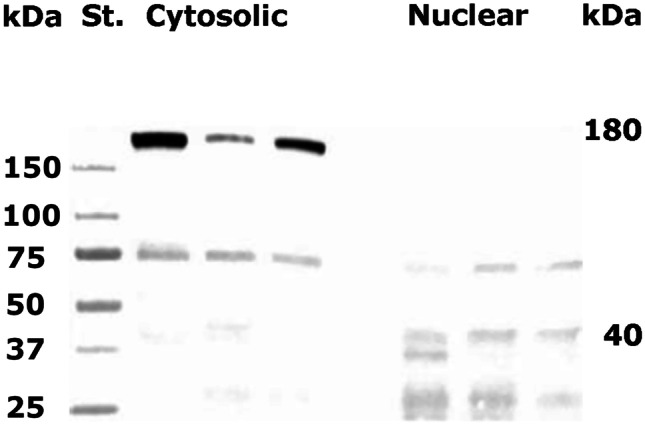
Western blot of cytosolic and nuclear fractions of honey bee fat body tissue. St. size standard. The full-length (180 kDa) Vg is dominating the cytosolic fraction of the fat body proteins, whereas the 40-kDa N-sheet mostly localizes in the nuclear fraction. Also, other fragments of approximately 70 and 25 kDa were visible in the nuclear fraction. Three individuals were pooled for each lane. *N* = 3.

We used *N* = 9 winter worker bees collected from two hives at Norwegian University of Life Sciences, Aas, Norway. Winter bees (i.e., *diutinus* bee*s*) have high levels of Vg and live extended lifespans when forage is unavailable and brood production ceases. The precise age of the bees was unknown, but winter bees can live 20 weeks or longer, surviving off food stores and fat reserves (Maurizio and Hodges 1950). Bees were anesthetized in cold, and the fat body tissue was dissected in ice-cold phosphate-buffered saline (PBS). Tissues were placed in three tubes with 50 µl hypotonic buffer (20 mM Tris–HCl pH 7, 10 mM NaCl, and 3 mM MgCl_2_) pooling three individuals per tube. A total of 2.5 µl 10% NP-40 was added, followed by 10-s vortex and centrifugation for 10 min 3000 g at 4 °C. The supernatant was collected as the cytosolic fraction. The pellet was washed once with 500 µl hypotonic buffer, and then suspended in 30 µl hypotonic buffer with 5 mM EDTA, 1% Tween-20, and 0.5% SDS and vortexed. All samples were then centrifuged for 10 min 15,000 g in 4 °C. Fifteen microliters of each sample was run on SDS-PAGE gel and blotted (Figure 1).

### Western blotting

The gel and Western blot reagents were purchased from Bio-Rad, and the protocol for all blots was as follows: SDS-PAGE-separated proteins were transferred to a nitrocellulose membrane. The blotted nitrocellulose membrane was incubated with PBS containing 0.5% Tween with 2.5% bovine serum albumin (BSA) overnight. The membrane was incubated for 1 h with the N-sheet antibody (1:2000) and for 1 h with a horseradish peroxidase-conjugated secondary antibody (1:5000) prior to imaging using an Immun-Star kit. All gels and blots were imaged, and band intensities were measured using a ChemiDoc XRS imager (Bio-Rad).

### Immunohistology

We used *N* = 6 winter worker bees. The gut was removed, and the abdomen detached and fixed overnight in 4% paraformaldehyde (PFA) in 1 × PBS at 4 ºC, followed by 3 × 10-min washes in PBS. The fat body was dissected in PBS and de/rehydrated with a full ethanol series. For each individual, one tissue sample was used as test (incubated with primary and secondary antibodies) and another one as control (no primary antibody incubation). The samples were incubated with the primary antibody overnight in 4 °C (1:50 in 2% BSA-PBS-Triton X-100). DAPI (Sigma-Aldrich [D9542] 1:1000) was used as a nuclear marker. The anti-rat secondary antibody was Alexa-568 nm that has no emission spectrum overlap with DAPI. For qualitative anatomical descriptions, a high NA (= high resolution) objective was chosen (40 × immersion oil; NA 1.25). Scans were taken with zoom 1.0 and zoom 2.0. All images were taken on a Leica TCS SP5 microscope in sequential acquisition mode to minimize crosstalk between the two channels for detecting DAPI and the Vg N-sheet signal.

### Whole Vg purification

Vg was purified from winter worker bee hemolymph as in Salmela et al. (2015).

### Labeled Vg in cell culture

Purified whole Vg was labeled with an Alexa Fluor 488 protein labeling kit (Invitrogen, Carlsbad, CA, USA). HighFive cells (i.e., a line of ovarian cells originating from the cabbage looper) were grown on an 8-well chamber slide (Thermo Fisher) overnight (number of slides one, repeated three times). The media was replaced with 20 µg/µl Vg-488 in PBS and incubated in the dark for 1 h. The cells were washed twice with PBS, fixed with 4% PFA, and washed twice again. DAPI was used as a nuclear marker. The cells were imaged the following day with Leica TCS SP5 MP (63 × objective).

### DNA-binding prediction

The following prediction tools available for protein-DNA binding were tested with default settings: sequence-based DNABIND (Liu and Hu 2013), DP-BIND (Hwang et al. 2007) and DRNApred (Yan and Kurgan 2017), and structure-based DNABIND (Liu and Hu 2013) and DISPLAR (Tjong and Zhou 2007). The structure used was the honey bee Vg N-sheet homology model published previously (Havukainen et al. 2011).

### ChIP-seq

To test empirically whether Vg N-sheet binds to DNA, and to determine what types of genes and genomic regions Vg N-sheet is bound to, we performed ChIP-seq on 1-day-old and 7-day-old worker bees. Newly emerged adult workers will take on tasks such as cleaning comb cells, while workers at 7 days old act as nurses to nourish the queen and developing larvae. Thus, workers in these two age groups span an important behavioral transition or maturation that is integral to a colony’s division of labor. To create samples, we pulled a brood frame from a single colony and placed it in an incubator overnight at 34 °C with 50% relative humidity to allow new adult bees to emerge. A subset of newly emerged bees was processed immediately (i.e., 1-day-old workers), while another subset was paint marked, returned to their source colony, and recollected 7 days later (i.e., 7-day-old workers). To process samples, we pooled freshly harvest fat body tissue from 10 individuals from each age group, flash froze them in liquid nitrogen, and homogenized them with a mortar and pestle.

The ChIP protocol was adapted from Bai et al. (2013), using Dynabeads™ Protein G (Invitrogen). Briefly, homogenized samples were cross-linked in 1% PFA in PBS, quenched with glycine (125 mM), washed in PBS with 1% protease inhibitors (Invitrogen), and then lysed in a cell lysis buffer containing NaCl (140 mM), HEPES (15 mM, pH 7.6), EDTA (1 mM), EGTA (0.5 mM), protease inhibitor (1%), Na-deoxycholate (0.1%), and sarkosyl (0.02%). The lysate was sonicated with a Branson 450 sonicator to reduce chromatin size to ~500 bp. To perform the immunoprecipitation, 100 μl of Dynabeads™ Protein G (Invitrogen) was first blocked with 0.5% BSA in PBS, and then the chromatin and anti-Vg antibodies were added to the mixture. The mixture was washed before being eluted in a buffer (Tris–HCL 50 mM, EDTA 1 mM, SDS 1%). Finally, the DNA was reverse cross-linked at 65 °C for 10 h and purified. We opted to use polyclonal antibodies raised in rabbits against the whole 180 kDa Vg molecule (Pacific Immunology, Ramona, CA) (Jensen and Børgesen 2000) rather than the rat-origin antibodies against the 40-kDa Vg N-sheet used elsewhere in this study because of their superior immunoprecipitation performance. In a preliminary study, the rabbit-origin whole-Vg antibodies consistently pulled down more chromatin than multiple batches of the rat-origin Vg N-sheet antibodies, which failed to retrieve sufficient chromatin for sequencing. This is likely due to Dynabeads™ Protein G (and A) having a greater affinity for rabbit-origin than rat-origin antibodies, as per the manufacturer. As a negative control, we compared immunoprecipitated DNA against input DNA (i.e., DNA from the same sample incubated with BSA-blocked Dynabeads™ but not precipitated with antibodies). We opted against using anti-GFP antibodies in our negative control, as this type of negative control is less commonly used and more prone to background noise (Xu et al. 2021).

Chromatin samples were sequenced at the DNASU lab at Arizona State University. The raw Illumina 2 × 75 bp pair-end reads were quality checked using FastQC v0.10.1 (Andrews 2010), followed by adapter trimming and quality clipping by Trimmomatic 0.35 (Bolger et al. 2014). Any reads with start, end, or the average quality within 4 bp window falling below quality scores 18 were trimmed. The clean reads were aligned to reference genome *Apis mellifera* Amel_4.5 (https://www.ncbi.nlm.nih.gov/genome/48?genome_assembly_id=22683) by Bowtie 2 version 2.2.9 (Langmead and Salzberg 2012). Another round of QC was performed on bam files. Library complexity was checked by NRF (non-redundancy fraction), defined as the number of unique start positions of uniquely mappable reads divided by number of uniquely mappable reads. All the samples passed the threshold 0.8 recommended by ENCODE. IGVtools and bamCompare from deepTools (Ramírez et al. 2014) were employed to compare two BAM files based on the number of mapped reads. First, the genome is partitioned into bins of equal size and then the number of reads in each bin is counted. The log2 value for the ratio of number of reads per bin was reported for IGV visualization. MACS2 was used for peaks calling with 0.01 FDR cutoff. Narrowpeak files as MACS2 output were annotated by HOMER (Heinz et al. 2010). It first determines the distance to the nearest transcription start site (TSS) and assigns the peak to that gene. Then, it determines the genomic annotation of the region covered by the center of the peak, including TSS, transcription termination site (TTS), exon, intronic, or intergenic.

To test for non-random occurrence of peaks within genome features, we used 1000 random peak datasets from the 7-day-old worker dataset. To create the random peak datasets, we used the shuffle program of the BEDTools package (Aronesty 2011) on the 782 peaks from the 7-day-old dataset that were located on full chromosome assemblies to generate 1000 bed files with peak locations that were shuffled within chromosomes, such that shuffled peak locations were non-overlapping and did not occur in assembly gaps. The annotatePeaks.pl program from the Homer package (Heinz et al. 2010) was then used to annotate the 1000 shuffled peak datasets and the 7-day-old peak dataset with respect to genome features in the NCBI *Apis mellifera* RefSeq annotation. We used chi-squared tests to determine whether the observed numbers of peaks overlapping with promoter regions (1 kb upstream to 100 bp downstream of the TSS), TTSs, exons, introns, and intergenic regions were greater or less than expected by chance.

### Gene ontology

We performed a gene ontology (GO) term analysis to determine if our list of Vg N-Sheet-DNA binding sites was enriched for any biological processes, molecular functions, cellular components, or KEGG pathways. We used the list of sites from 7-day-old bees, but omitted sites found in *intergenic* regions. Honey bee genes were converted to *D. melanogaster* orthologs (*N* = 360 genes), as this latter genome is more robustly annotated. We used DAVID 6.8 (Huang et al. 2009) with default settings, and the resulting terms are those deemed significant (*P* < 0.05) after Benjamini–Hochberg corrections.

### Vg N-sheet-DNA binding motif search

For de novo motif identification, we created a non-redundant dataset of 790 peaks by combining peaks identified in the 1-day-old and 7-day-old samples and merging peaks with overlapping regions between the two datasets. We used GimmeMotifs (Heeringen et al. 2011) which ran and combined results for ten motif prediction packages – Mdmodule, Weeder, GADEM, trawler, Improbizer, BioProspector, Posmo, ChIPMunk, JASPAR, AMD, HMS and Homer. GimmeMotifs clusters the results, performs enrichment, and computes receiver operator characteristic (ROC) and mean normalized conditional probability (MNCP). Half of the peaks (395) were used to train each algorithm, and the other half was used for testing. Since we did not have *a priori* knowledge of the motif length, we ran GimmeMotifs four times with different size range options (small 5–8 bp, medium 5–12 bp, large 6–15 bp, xl 6–20 bp). Since the peaks were located throughout the genome, we used sequences randomly chosen from portions of the genome with similar GC content to the peak sequences as the background for the enrichment tests.

### Vg response to immune challenge, enzymatic cleavage, and 3D structural model

See S4.

## Results

### Vg translocation to the nucleus

#### Cell fractionation

To verify the nuclear location of the Vg N-sheet, we separated fat body cells into cytosolic and nuclear components by cell fractionation followed by Western blot. Full-length Vg (180 kDa) and a ~ 75-kDa band were found in the cytosolic component, while the nuclear component only contained fragments below 75 kDa, including the 40 kDa Vg N-sheet (Figure 1). We have shown previously that the 40-kDa Vg N-sheet is a specific cleavage product of fat body cells and not simply a biproduct of unspecific degradation (Havukainen et al. 2011, 2012). We also observed 70, 37, and 25 kDa fragments in the nuclear fraction, but these are likely degradation products caused by the method, as the bands are faint or non-existent in untreated Western blot samples (see S1).

#### Immunohistology

**Figure 2.**
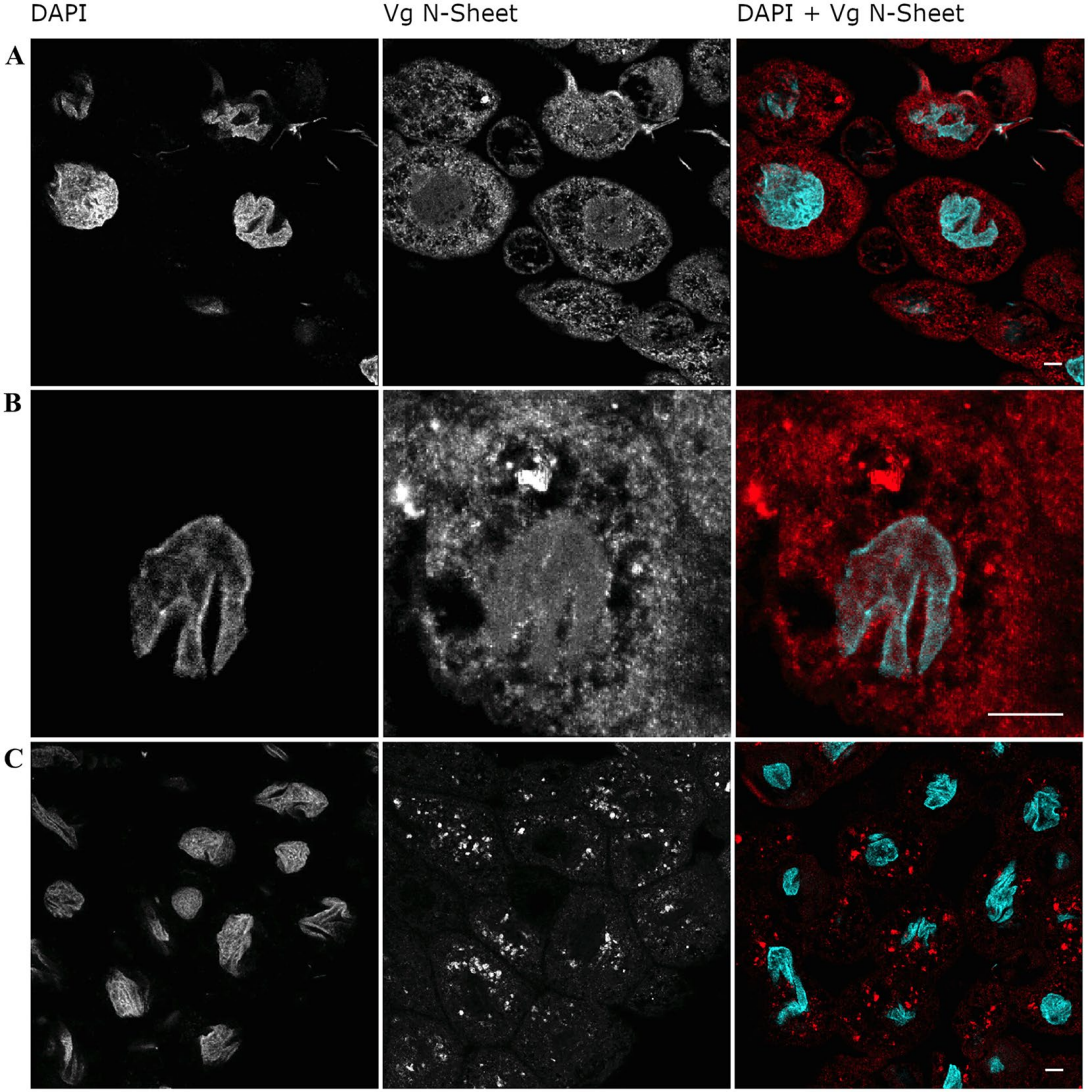
Localization of V signals in the honey bee fat body using an antibody targeting the Vg N-sheet. **A**–**C** Confocal images of fat body cells representing six biological samples. The left panel depicts the nuclear stain DAPI, the middle panel depicts Vg N-sheet signal via binding of the secondary antibody (Alexa 568), and the right panel depicts the superposition of DAPI (cyan) and Vg N-sheet signal (red). **A** Vg N-sheet signal co-localizes with DAPI in cell nuclei. **B** Zoom-in of a single cell showing Vg N-sheet nuclear translocation. **C** Cells that do not show Vg N-sheet co-localization with DAPI. Instead, Vg signal is found in granules in the cytosol. The scalebar = 10 µm, and magnification = 40 × objective. Control staining images (no primary antibody) are displayed in S2.

We observed fat body cells using confocal microscopy (Figure 2A–C). Honey bee fat body contains two cell types: trophocytes are characterized by irregularly shaped nuclei and are responsible for Vg production and storage, while oenocytes have spherical nuclei and do not produce or contain Vg (Pan et al. 1969; Roma et al. 2010). We observed two Vg localization patterns in trophocytes: (i) Vg signal co-localized with DAPI in the nucleus, and also found spread throughout the cytosol (Figure 2A, B), and (ii) Vg signal absent in the nucleus and instead restricted to granule-like formations in the cytosol (Figure 2C, see also Havukainen et al. 2011 for observations of this pattern). Controls for unspecific secondary antibody staining were included (S2 A-B), which indicated that the immunosignals present in Figure 2A–C are specific for Vg N-sheet primary antibodies.

#### Labeled whole Vg in cell culture

**Figure 3.**
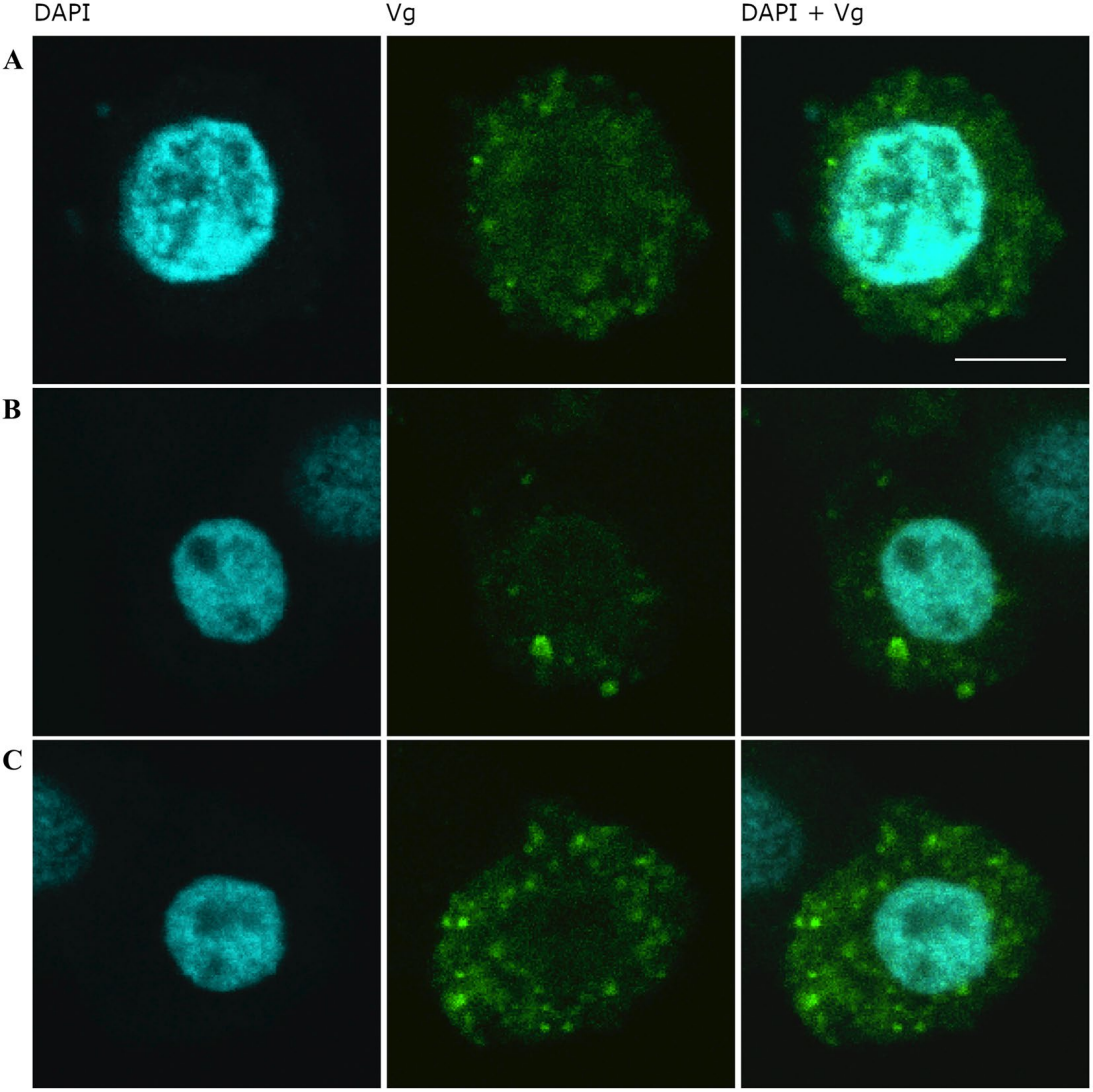
Insect ovarian HighFive cells cultured with honey bee Vg. HighFive cells were incubated with purified honey bee Vg labelled with an Alexa 488 fluorophore. After 1 h incubation, Vg was observed both in the cytosol as bright granules and in the nucleus as a haze, indicating uptake of Vg into the nucleus. Scalebar = 5 µm, and magnification 63 × objective. Representative negative controls (samples not incubated with Vg) are displayed in S3.

To rule out that the nuclear signal was an artefact caused by Vg N-sheet primary antibody, we incubated cell cultures (Lepidopteran ovarian HighFive cells) with antibody-free pure fluorescently labeled Vg. During incubation, the fluorescent Vg was imported into cells and was visible in the cell nuclei co-localizing with DAPI, and also visible in the cytosol in granule-like formations (Figure 3, control images S3).

### Vg N-sheet-DNA binding

#### *In silico *binding prediction

Using the whole Vg amino acid sequence, two separate programs with different search algorithms, DP-Bind (Hwang et al. 2007) and DRNApred (Yan and Kurgan 2017), both identified the same amino acid residue stretch as a putative DNA binding domain: SRSSTSR in position 250–256 of the N-sheet domain of Vg. Another sequence-based program, DNABIND (Liu and Hu 2013), also identified the Vg N-sheet as a DNA-binding protein. This program predicts a protein’s DNA binding probability and sets a threshold probably of 0.5896, above which a protein is statistically likely to be able to bind DNA. The Vg N-sheet scored a 0.6267 probability, indicating it can bind DNA. Additionally, the structure-based prediction software DISPLAR (Tjong and Zhou 2007) identified the SRSSTSR stretch as capable of binding DNA using a published honey bee Vg N-sheet model (Havukainen et al. 2011) as input. There were another 12 and 3 additional stretches identified by DP-BIND and DRNApred, respectively, which did not overlap between the programs, whereas DISPLAR identified two additional stretches that did not overlap with predictions made by the two sequence-based programs. Taken together, results from multiple prediction software platforms support the hypothesis that Vg can bind to DNA, and that this capability is likely restricted to the N-sheet.

#### ChIP-seq

**Figure 4.**
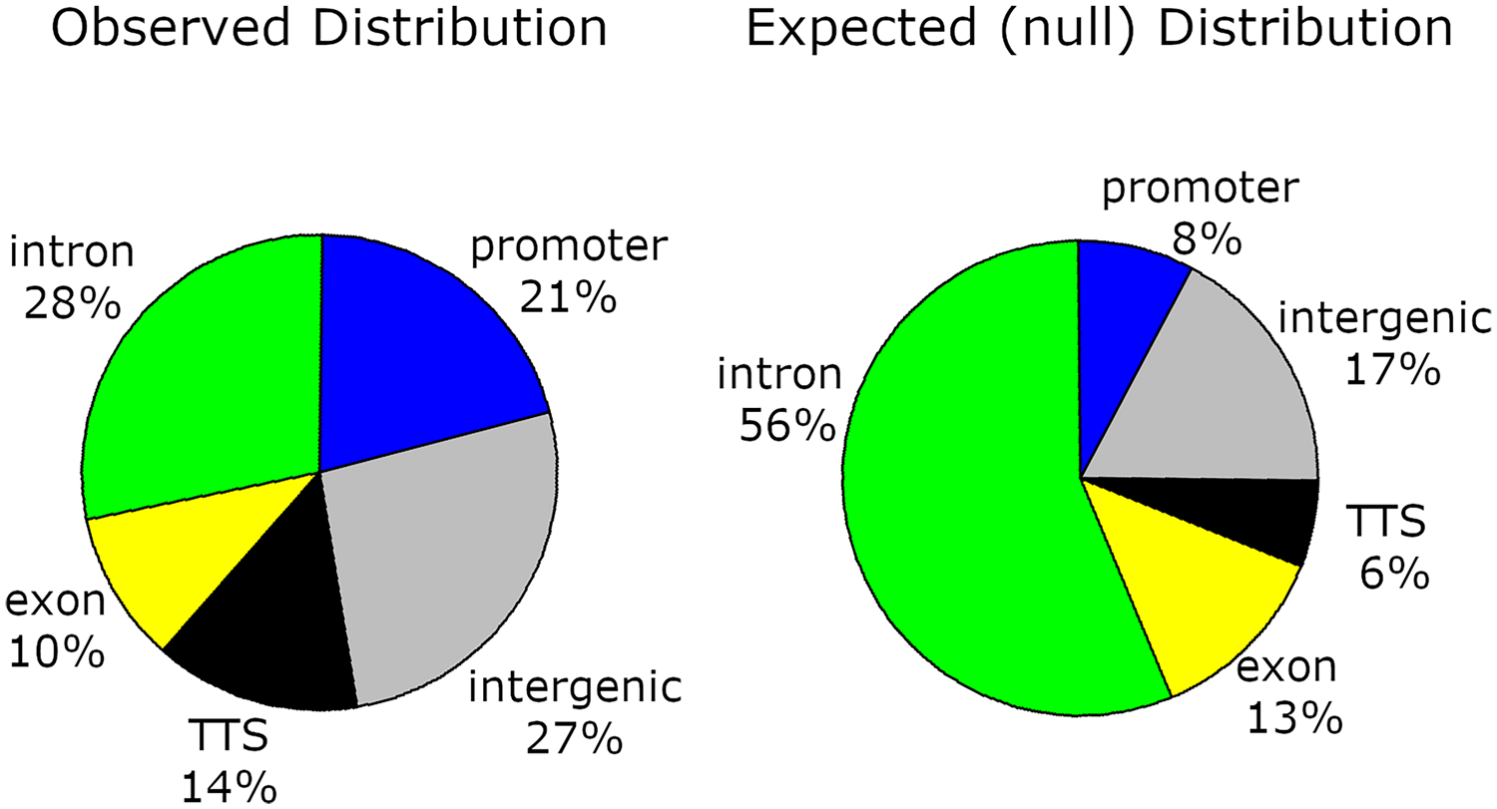
Genomic distribution of observed Vg N-sheet-DNA binding sites compared with a null distribution. The null distribution was created by randomly shuffling 782 non-overlapping ChIP-seq peaks for 1000 iterations. We found more Vg N-sheet-DNA binding sites than expected by chance in promotor regions (*χ*^2^ = 175.18, *P* = 5.47E − 40), transcription termination sites (*χ*^2^ = 91.68, *P* = 2.09E − 24), and intergenic regions (*χ*^2^ = 46.14, *P* = 1.10E − 11), and fewer than expected by chance in exons (*χ*^2^ = 3.75, *P* = 0.05) and introns (*χ*^2^ = 244.55, *P* = 4.01E − 55).

ChIP DNA had significant enrichment (FDR < 0.01) at 90 and 782 putative Vg N-sheet-DNA binding sites on fully assembled chromosomes in 1-day-old and 7-day-old workers, respectively. Of the 90 binding sites in 1-day-old bees, 83 (92%) were also present in 7-day-old bees, illustrating an expansion of Vg N-sheet-DNA binding sites as workers age. Using the 7-day-old worker binding sites, we analyzed their genomic distribution compared to a null distribution of random peaks and found a greater number than expected by chance within promotor regions, TTSs, and intergenic regions, and fewer than expected by chance in exons and introns (Figure 4).

#### Gene ontology

Next, we again used the 7-day-old worker binding sites to perform a gene ontology (GO) term analysis. We omitted binding sites located in intergenic regions and restricted our list to sites located in or near genes (promotor regions, introns, exons, TTSs, *N* = 574 binding sites across 484 genes). We converted these *Apis mellifera* genes into *Drosophila melanogaster* orthologs (*N* = 274 genes) using the Hymenoptera Genome Database (Elsik et al. 2016) and used DAVID Bioinformatics Resources (Huang et al. 2009) to look for enrichment of biological processes, molecular functions, cellular components, and KEGG pathways. We found significant enrichment (Benjamini-Hochberg-corrected *P* value < 0.05) for 142 terms and pathways (see Table S1). There was much overlap in these terms, but most genes had functions pertaining to *developmental processes* (GO:0,032,502, *N* = 134 genes, adj. *P* = 6.58 E − 8), *neurogenesis* (GO:0,022,008, *N* = 70 genes, adj. *P* = 4.63 E − 8), *signaling* (GO:0,023,052, *N* = 75 genes, adj. *P* = 4.74 E − 5), or similar functions. Many genes bound by the Vg N-sheet code for membrane-bound receptors that are known to play roles in complex phenotypes, like behavior, including receptors for corazonin, glutamate, acetylcholine, serotonin, and octopamine (see Table S1 for complete list of Vg N-sheet binding sites). Furthermore, the Vg N-sheet was bound to more than a dozen immunological genes, including *Defensin-1*, a key antimicrobial peptide, and *sickie*, an integral component of the Immune Deficiency pathway defense against gram-negative bacteria. These results demonstrate that Vg N-sheet binds to DNA at hundreds of loci in the honey bee genome, that many are behaviorally, developmentally, and immunologically relevant, and that many of these Vg N-sheet-DNA interactions persist as adult workers age and transition between tasks.

#### Binding motif identification

**Figure 5.**
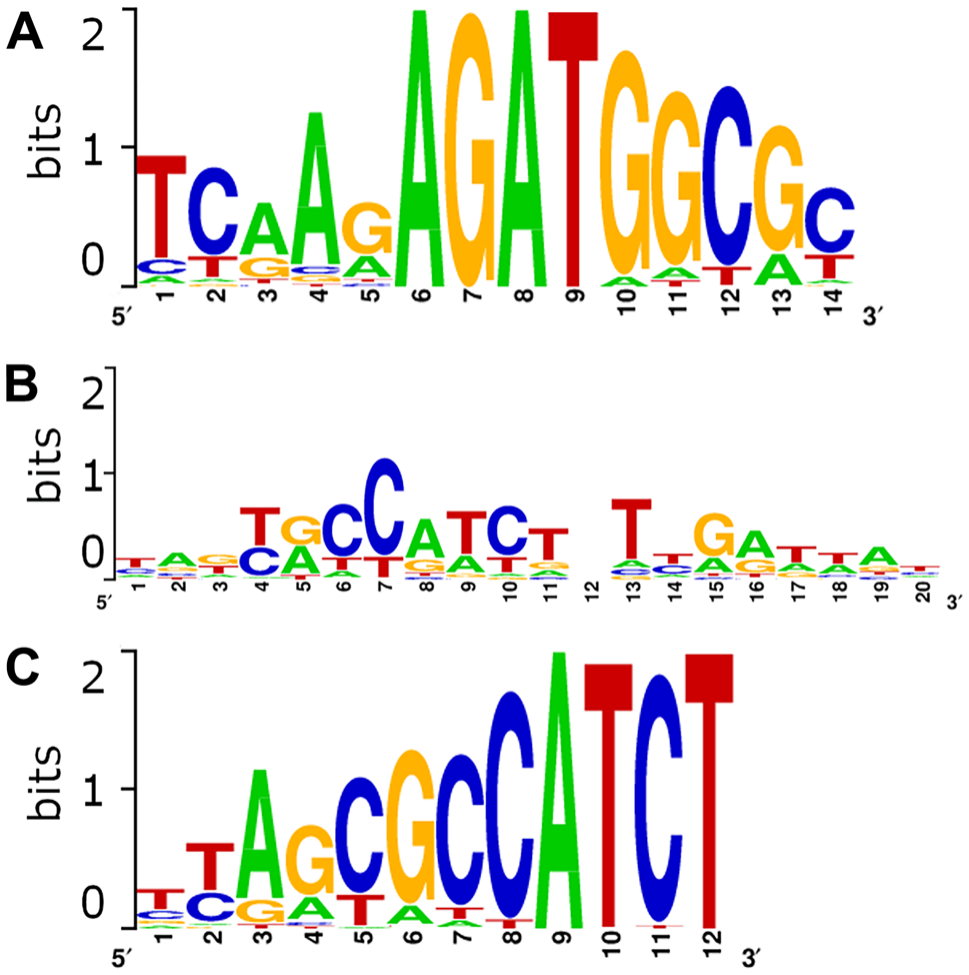
Logo representations for the top motif predictions. **A** Top motif prediction from analysis with “large” motif setting (35.75% enrichment). **B** Top motif prediction from analysis with “medium” motif setting (34.5% enrichment). **C** Top motif prediction from analysis with “xl” motif setting (33.1% enrichment).

We analyzed the binding site data to look for DNA sequences to which the Vg N-sheet may be binding. We ran four analyses using GimmeMotif (Heeringen et al. 2011), and we selected the top motif predictions, based on a combination of percent enrichment, *P*-value, ROC-AUC, and MNCP (Table I) (Figure 5). The top motif prediction for the small motif run was a sub-motif of the large motif run, so it was discarded. These results suggest that there are specific sequence motifs to which the Vg N-sheet binds.

**Table I:** Top de novo motif predictions. The top results for medium (5–12 bp), large (6–15 bp), and extra-large (6–20 bp) motifs were generated using GimmeMotif. Included are the number of peaks containing the given motif, and percent enrichment compared to background (false positive rate 1%), and the enrichment *p*-value (hypergeometric/Fisher’s exact). Also included are the area under the receiver-operator characteristic curve (ROC-AUC), the mean normalized conditional probability (MNCP), the best known matches to other transcription factors, and the *p*-value for these matching transcription factors

Motif	Analysis	No. peaks with motif	Enrichment	*P*-value	ROC-AUC	MNCP	Best known match	*P*-value for Match
nAkyrCCATCTnTyGrwwAn	Medium	310	34.5	2.12E − 116	0.788	5.58	bHLH_Average_34	2.70E − 02
TCAAGAGATGGCGC	Large	300	35.8	6.36E − 122	0.737	5.44	C2H2_ZF_M2196_1.01	5.27E − 04
TyAGCGCCATCT	xl	314	33.1	2.81E − 116	0.725	5.24	C2H2_ZF_M2196_1.01	8.10E − 07

### Vg cleavage dynamics

#### Vg protein cleavage and nuclear translocation in response to immune challenge

We found that Vg incubated in vitro with *E. coli* had increased protein fragmentation, but that worker bees that ingested *E. coli* possessed fewer fat body nuclei with Vg signal within than workers fed a control diet (see S4).

#### Vg structure and enzymatic cleavage

**Figure 6.**
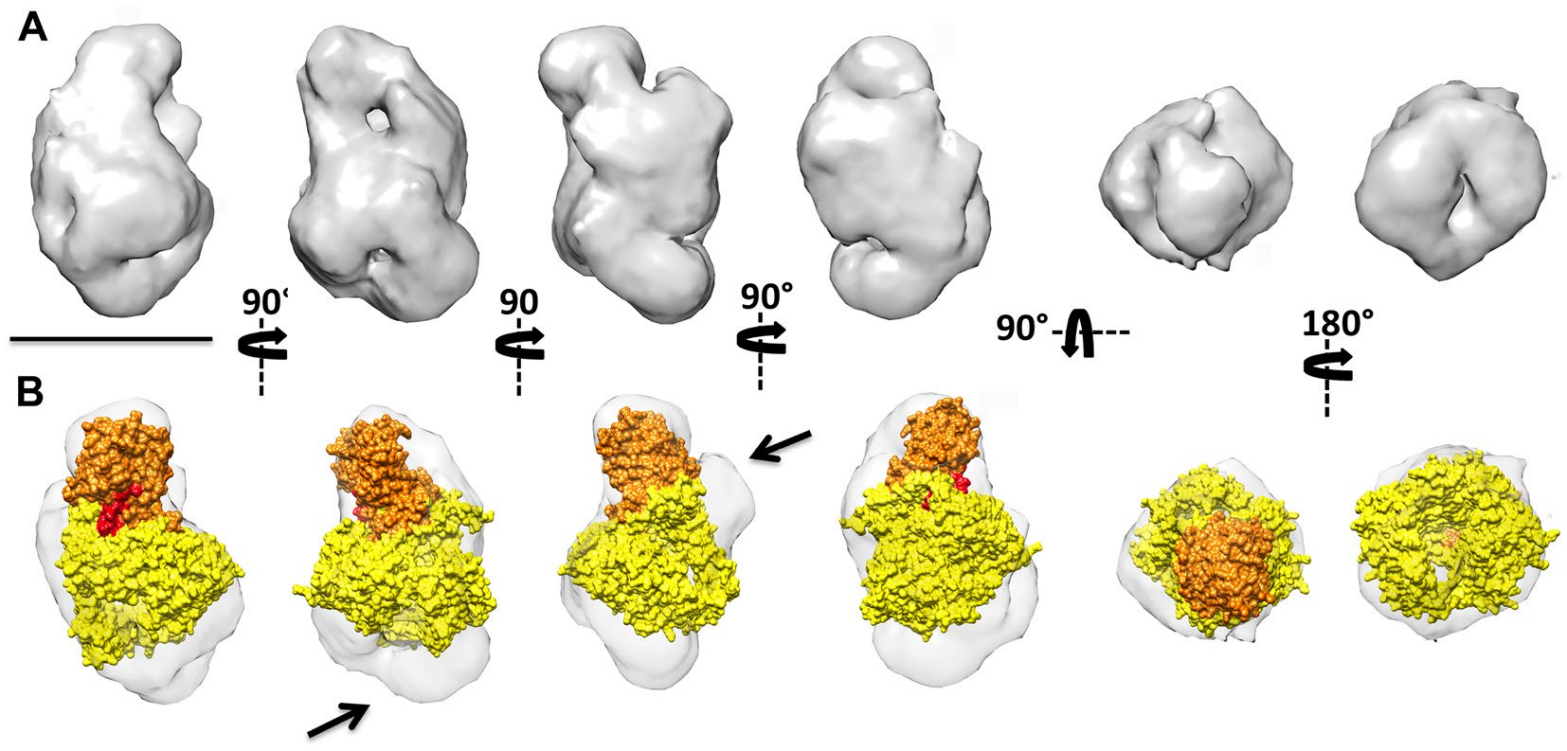
The 3D reconstruction of the honey bee Vg reveals the exposure of the cutting site. **A** Different views of the 3D reconstruction of the Vg from honey bee. The four on the left are orthogonal side views of the volume, whereas the two on the right correspond to the two end-on views of the 3D reconstruction. Bar indicates 100 Å. **B** The same views with the atomic structure of lipovitellin from lamprey (pdb 1lsh) docked into the EM 3D reconstruction. The N-sheet domain, which protrudes from the main body of the structure, is highlighted in orange. The domain colored red points to the linker that connects the N-sheet domain to the lipid cavity (yellow mass).

We found that the linker region connecting the N-sheet with the rest of the Vg molecule protrudes from the structure, providing easy access to proteolytic enzymes (Figure 6). Moreover, enzymatic cleavage is likely carried out, at least partially, via dephosphorylation and caspase activity (see S4).

## Discussion

Vg dates back at least 700 million years to the time when animals first appeared (Hayward et al. 2010) (S5), and the ubiquity of its roles in reproduction and immunity across diverse taxa (Du et al. 2017; Li et al. 2008; Salmela et al. 2015) suggests that these functions developed early in its evolutionary history. Several of its structural domains are highly conserved, and yet the molecular mechanism by which Vg regulates so many complex traits like behavior and longevity has remained something of a mystery. Here, we reveal a major and hitherto unknown ability of Vg that may provide such a molecular explanation: nuclear translocation and DNA binding. Vg may be directly involved in modulating gene expression, and given its prevalence in the animal kingdom and its conserved molecular structure, it is possible that it performs a similar function across a wide array of oviparous animals.

In our study, we found strong evidence of the Vg N-sheet translocating to the nucleus using three methods: Western blot of proteins in the fractionated nuclear component, immunohistology, and antibody-free localization of Vg in cell culture. The two first approaches confirmed the Vg N-sheet domain to be present in both nucleus and cytosol, with the former result reaffirming previous findings (Havukainen et al. 2011). Moreover, the first approach specifies that the cell nucleus excludes the full-length 180 kDa Vg molecule and the previously reported specific Vg fragmentation product of 150 kDa size (i.e., the Vg molecule without the N-sheet domain) (Havukainen et al. 2011). The cell culture approach makes it less likely that antibody artefacts explain the outcomes of the first experiments.

We found theoretical support for Vg’s DNA binding ability using several prediction software platforms, and this prompted us to test this ability empirically. Our ChIP-seq analysis confirmed that the honey bee Vg N-sheet binds to many loci in fat body DNA of 1-day-old and 7-day-old workers. Despite there being far fewer Vg N-sheet-DNA binding sites in 1-day-old than 7-day-old workers (150 vs 927 loci, respectively), there is robust overlap in binding sites between these two age groups as over 90% of sites observed in 1-day-old workers are still present in 7-day-older workers. This suggests that the Vg N-sheet not only maintains long-term associations with specific loci, but also that the repertoire of binding sites expands as workers age and behaviorally transition from cell cleaning to nursing.

GO term analysis of Vg N-sheet-DNA binding sites hint at the types of biological functions that Vg may be targeting for regulation. We found significant enrichment for the GO terms *developmental processes* (GO:0,033,502), *neurogenesis* (GO:0,022,008), and *signaling* (GO:0,023,052), among many others, and many of the genes included within these terms code for membrane proteins. Indeed, many genes code for G protein-coupled receptors and ion channels (Table S1), suggesting that Vg regulates components of signal transduction pathways. Interestingly, 1-day-old and 7-day-old workers perform different tasks, and many of these receptors are known to affect how individuals behave and respond to stimuli. These include receptors for glutamate (Kucharski et al. 2007), acetylcholine (Eiri and Nieh 2012), corazonin (Gospocic et al. 2017), and octopamine (Grohmann et al. 2003). One possible explanation is that as workers age and transition between tasks, they respond to different signals from nestmates and the environment, and thus must be equipped with relevant molecular machinery to receive and respond to those signals.

A potential shortcoming here is that these Vg N-sheet-DNA binding loci are from fat body cells rather than neurons, so the effect on honey bee behavior might appear limited. However, Vg is neither found nor expressed in honey bee neurons (Münch et al. 2015), limiting its likelihood of binding to DNA therein. Fat body, on the other hand, not only produces and stores Vg, but also plays a central role in regulating metabolism and homeostasis. In this capacity, it produces many products that affect a wide range of insect behaviors, including courtship (Lazareva et al. 2007), host-seeking (Klowden et al. 1987), and onset of foraging (Antonio et al. 2008). Moreover, the fat body is a key player in innate immunity (Fehlbaum et al. 1994; Lycett et al. 2006; Morishima et al. 1997), and our data show Vg N-sheet to be bound to several important immune-related genes in this tissue. These include *defensin-1*, an important antimicrobial peptide (Ganz 2003), *sickie*, a regulator of immune transcription factors (Foley and O’Farrell 2004), and several genes involved in autophagy and phagocytosis (Levine and Deretic 2007; Strand 2008) (Table S1). Taken together, the fat body’s central role in honey bee biology means that any protein-DNA binding herein could have wide-ranging effects on numerous physiological pathways. This work also revealed several putative de novo DNA binding motifs for the Vg N-sheet. The aim of this ChIP-seq work here was to determine whether Vg binds to DNA, and if so, at which loci. Our next step is to further our understanding of Vg’s actions inside the nucleus and to determine how it regulates gene expression across different caste types and populations in honey bees. This is a multi-faceted approach that will involve performing ChIP-seq on workers, drones, and queens from multiple colonies to determine how Vg N-sheet differentially binds to DNA, and performing gene expression analyses such as RNA-seq to elucidate how Vg N-sheet-DNA binding up or downregulates gene expression at various loci. Furthermore, we can distinguish whether Vg is a transcription factor or co-regulator by determining what other proteins it interacts with in the nucleus via co-immunoprecipitation and mass spectrometry (Li et al. 2016). This will greatly enhance our knowledge of Vg’s regulatory properties and can potentially unveil co-evolutionary relationships between Vg and other proteins. Nevertheless, the findings presented in this study here represent a major new discovery of Vg function.

Our discovery that the Vg N-sheet subunit binds to DNA in fat body cell nuclei raises several questions that warrant further research. First, does Vg translocate into the nucleus in other tissues in addition to the fat body? Vg has been verified in eggs and ovaries (Seehuus et al. 2007), in hypopharyngeal glands (Seehuus et al. 2007), in immune cells (Hystad et al. 2017), and in glial cells of the honey bee brain (Münch et al. 2015). In this latter observation, it is specifically the Vg N-sheet that is localized in glial cells, and such subcellular localization should prompt a highly relevant research subject since honey bee *vg* knock-downs show an altered brain gene expression pattern (Wheeler et al. 2013) and major behavioral changes (Nelson et al. 2007). Second, does Vg naturally translocate to the nucleus in other animals? The Vg N-sheet cleavage pattern is similar in most insects studied (Tufail and Takeda 2008), but the possibility of N-sheet translocation remains speculative before it is experimentally tested in another species. Finally, what role do other Vg fragments play in nuclear translocation? The N-sheet-specific antibodies, as well as Vg purified from fat body, show weak protein bands smaller in size to full-length Vg, most notably, bands of ~ 75 and ~ 125 kDa in size. It is unclear if these are functional Vg fragments or simply the result of unspecific fragmentation. In general, we have observed that Vg fragment number grows in harsh sample treatment conditions. We suspect that at least a 25-kDa fragment detected by the Vg antibody used here in our tissue fractioning assay is a degradation product caused by the assay protocol. However, it is not ruled out that other Vg fragments in addition to N-sheet play a role in nuclear localization of Vg.

Vg was first discovered as an egg-yolk protein, but the protein is so ancient that we cannot be certain of its original function, and it is possible that it assumed a gene regulatory role via DNA-binding early in its history. While we only touch on the mechanistic link between Vg and its multifunctionality, our results will hopefully spark diverse future studies on the role of Vg as a putative transcription factor in honey bees and other animal species.

## Supplementary information

Below is the link to the electronic supplementary material.

Supplementary file1 (PDF 29 KB)

Supplementary file2 (PDF 128 KB)

Supplementary file3 (PDF 60 KB)

Supplementary file4 (PDF 231 KB)

Supplementary file5 (PDF 208 KB)

Supplementary file6 (XLSX 367 KB)

## Data Availability

All relevant experimental data are included in the manuscript and supplementary materials. 3D structural model of Vg is deposited in the Worldwide Protein Data Bank (D_1000249604).
