# Supplementary material for: Nuclear translocation of vitellogenin in the honey bee (Apis mellifera)
Source: Apidologie. 2022 Mar 15;53(1):13. doi: 10.1007/s13592-022-00914-9 (PMC8924143; doi:10.1007/s13592-022-00914-9)
Supplement: Supplementary file 1 — Supplementary file1 (PDF 29 KB) [file 13592_2022_914_MOESM1_ESM.pdf]

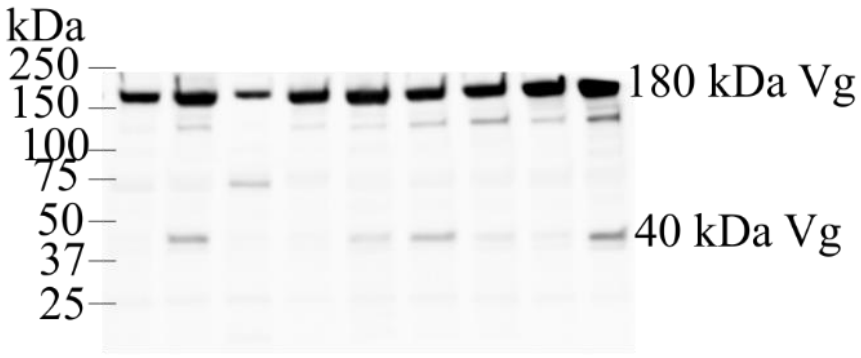

Fig S1. Western blot of vitellogenin N-terminal antibody (targeting amino acids 24-360) in honey bee tissue lysate samples. Abdomen of nine winter worker honey bee individuals were homogenized and western blotted (20 µg total protein per lane). There is a strong full-length vitellogenin band (180 kDa) in each sample. In addition, there are shorter fragments, whose presence and strength varies in individual samples. The previously identified 40 kDa N-terminal vitellogenin fragment is indicated. The other fragments of unknown function detected by this antibody in some individuals are ~75 kDa and ~125 kDa.
