# Supplementary material for: Nuclear translocation of vitellogenin in the honey bee (Apis mellifera)
Source: Apidologie. 2022 Mar 15;53(1):13. doi: 10.1007/s13592-022-00914-9 (PMC8924143; doi:10.1007/s13592-022-00914-9)
Supplement: Supplementary file 2 — Supplementary file2 (PDF 128 KB) [file 13592_2022_914_MOESM2_ESM.pdf]

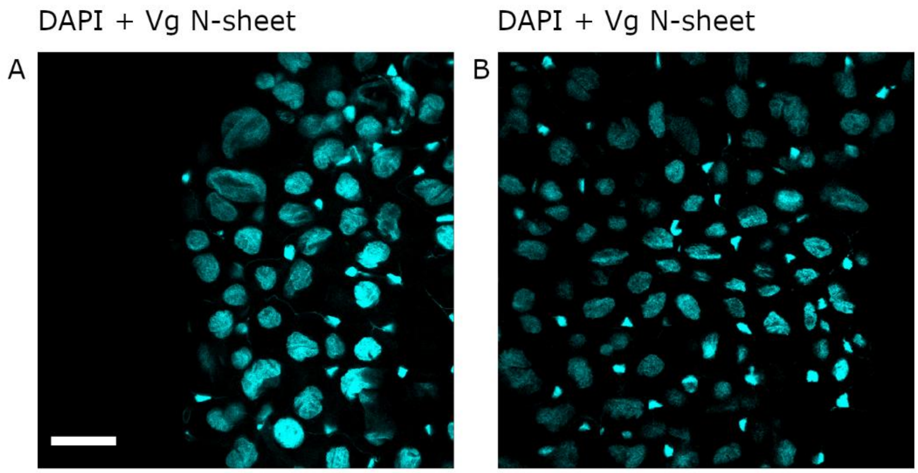

S2: Staining control for Fig 2. A-B depict separate biological replicates of honey bee fat body tissue that underwent negative control staining depicted in Fig 2. These samples were incubated *without* the primary antibody for the Vg N-sheet, but *with* the secondary antibody (Alexa 568). Here, the nuclear stain DAPI (cyan) is visible, while the Vg N-sheet stain (red) is absent, indicating that the secondary antibody is specific to the Vg N-sheet antibody. Scalebar = 50 μm.
