# Supplementary material for: Nuclear translocation of vitellogenin in the honey bee (Apis mellifera)
Source: Apidologie. 2022 Mar 15;53(1):13. doi: 10.1007/s13592-022-00914-9 (PMC8924143; doi:10.1007/s13592-022-00914-9)
Supplement: Supplementary file 3 — Supplementary file3 (PDF 60 KB) [file 13592_2022_914_MOESM3_ESM.pdf]

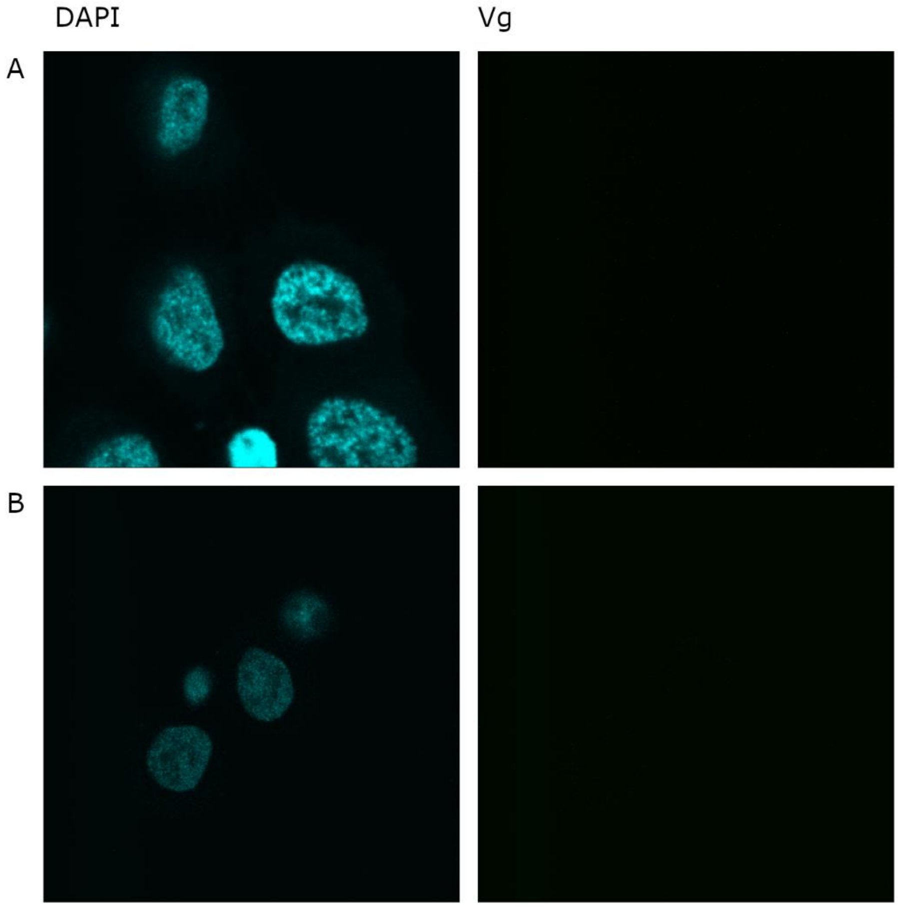

S3: Control images for Fig 3. A-B depict separate biological replicates. In the treated samples (Fig 3), insect HighFive cells were incubated with purified Vg labelled with Alexa 488 and cell nuclei were stained with DAPI. Here, HiveFive cells were *not* incubated with Vg labelled with Alexa 488. DAPI nuclear stain is visible in the left side panels, but in the right side panels no fluorescence is detected in the 488 wavelength. This demonstrates there is no autofluorescence in the 488 wavelength that could be misinterpreted as the Vg signal.
