# Supplementary material for: Nuclear translocation of vitellogenin in the honey bee (Apis mellifera)
Source: Apidologie. 2022 Mar 15;53(1):13. doi: 10.1007/s13592-022-00914-9 (PMC8924143; doi:10.1007/s13592-022-00914-9)
Supplement: Supplementary file 5 — Supplementary file5 (PDF 208 KB) [file 13592_2022_914_MOESM5_ESM.pdf]

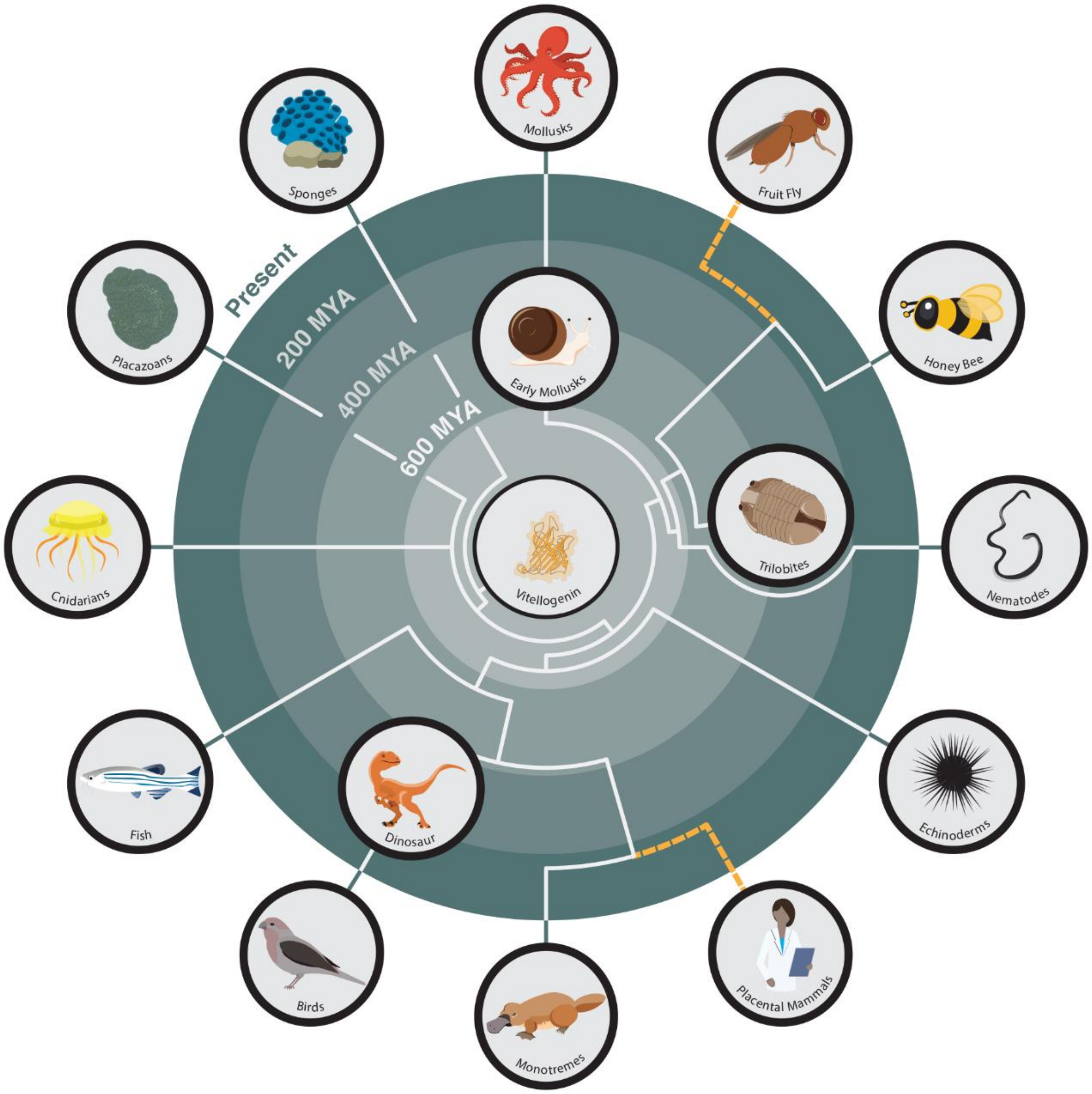

Fig 6. The deep phylogenetic history of Vitellogenin. Vg first evolved around 700 million years ago when Metazoans appeared (Hayward et al., 2010) and is present in all extant Metazoan phyla, from earliest animals like sponges and cnidarians to the more recently evolved chordates, like fish, birds, and monotreme mammals (Agnese et al., 2013; Akasaka et al., 2013; Babin, 2008; Biscotti et al., 2018; Chen et al., 2018, 1997; García-Alonso et al., 2006; Prowse and Byrne, 2012; Riesgo et al., 2014). Vg has been lost in several scientifically important lineages, including placental mammals and higher dipterans like *D. melanogaster* (depicted with dashed yellow lines) (Brawand et al., 2008; Sappington, 2002). Vg's earliest known functions pertain to egg-yolk formation and immunity, but it remains to be seen when DNA binding evolved.
